# An epithelial-to-mesenchymal transition induced extracellular vesicle prognostic signature in non-small cell lung cancer

**DOI:** 10.1038/s42003-022-04350-4

**Published:** 2023-01-18

**Authors:** Richard J. Lobb, Kekoolani S. Visan, Li-Ying Wu, Emma L. Norris, Marcus L. Hastie, Sarah Everitt, Ian A. Yang, Rayleen V. Bowman, Shankar Siva, Jill E. Larsen, Jeffrey J. Gorman, Michael MacManus, Antoine Leimgruber, Kwun M. Fong, Andreas Möller

**Affiliations:** 1 Tumour Microenvironment Laboratory, QIMR Berghofer Medical Research Institute, Herston, QLD 4006 Australia; 2 Faculty of Medicine, University of Queensland, Brisbane, QLD 4072 Australia; 3 School of Biomedical Sciences, Faculty of Health, Queensland University of Technology, Brisbane, QLD 4001 Australia; 4 Protein Discovery Centre, QIMR Berghofer Medical Research Institute, Herston, QLD 4006 Australia; 5 Department of Radiation Oncology, Peter MacCallum Cancer Centre, Melbourne, VIC 3000 Australia; 6 UQ Thoracic Research Centre, The University of Queensland, Brisbane, QLD 4072 Australia; 7 The Prince Charles Hospital, Brisbane, QLD 4032 Australia; 8 Sir Peter MacCallum Department of Oncology, The University of Melbourne, Melbourne, VIC 3000 Australia; 9 Oncogenomics Laboratory, QIMR Berghofer Medical Research Institute, Herston, QLD 4006 Australia; 10 Swiss Medical Network, Genolier, VD 1272 Switzerland; 11 Present Address: Department of Otorhinolaryngology, Chinese University of Hong Kong, Shatin, Hong Kong

**Keywords:** Prognostic markers, Non-small-cell lung cancer, Non-small-cell lung cancer

## Abstract

Despite significant therapeutic advances, lung cancer remains the leading cause of cancer-related death worldwide^1^. Non-small cell lung cancer (NSCLC) patients have a very poor overall five-year survival rate of only 10–20%. Currently, TNM staging is the gold standard for predicting overall survival and selecting optimal initial treatment options for NSCLC patients, including those with curable stages of disease. However, many patients with locoregionally-confined NSCLC relapse and die despite curative-intent interventions, indicating a need for intensified, individualised therapies. Epithelial-to-mesenchymal transition (EMT), the phenotypic depolarisation of epithelial cells to elongated, mesenchymal cells, is associated with metastatic and treatment-refractive cancer. We demonstrate here that EMT-induced protein changes in small extracellular vesicles are detectable in NSCLC patients and have prognostic significance. Overall, this work describes a novel prognostic biomarker signature that identifies potentially-curable NSCLC patients at risk of developing metastatic NSCLC, thereby enabling implementation of personalised treatment decisions.

## Abstract (web summary)

EMT-induced protein changes in small extracellular vesicles are detectable in NSCLC patients and have prognostic significance.

## Introduction

Lung cancer is the most common cause of cancer-related mortality worldwide and has one of the lowest survival outcomes of any cancer^2^. Most lung cancer cases are categorized as non-small cell lung cancer (NSCLC), and overall, NSCLC patients have an ~10–20% 5-year survival rate^2^. Tumour, node, metastasis (TNM) staging is currently the most important factor for predicting survival and guiding clinical interventions in NSCLC patients^3^. However, a significant proportion of potentially curable, locoregionally confined NSCLC patients have therapy-refractory disease or rapidly develop metastatic lesions despite curative-intent treatment^4^. This suggests that TNM staging alone is insufficient in guiding disease management. Therefore, there is a clinical demand to identify patients who respond poorly to current treatments, allowing for tailored treatment interventions.

Experimental and clinical studies have verified hypoxia as a driver of cancer progression and metastasis^5^. Within the tumour microenvironment, hypoxia alters numerous physiological functions, including angiogenesis, metabolism, immune cell function, and epithelial-to-mesenchymal transition (EMT)^5^, thereby promoting metastasis. EMT is pivotal in the metastatic cascade and involves the phenotypic depolarisation of epithelial cells into elongated, mesenchymal cells^6,7^. As such, EMT leads to enhanced cell invasion, migration, altered tumour microenvironment immune cell composition^8^, and resistance to apoptotic stimuli, such as chemotherapy^6,9^. Identifying biomarkers that are capable of detecting EMT in NSCLC would serve as a potential risk stratification tool in potentially curable NSCLC patients, facilitating improved clinical outcomes.

Prognostic biomarkers, in particular non-invasive liquid biomarkers, such as small extracellular vesicles (sEVs, also known as exosomes^10,11^), could allow clinicians to identify patients who may benefit from intensification of treatment or adjuvant treatment interventions. Small EVs are secreted, membrane-enclosed vesicles with a size-range of 30–150 nm in diameter^12^. As a specific subset of extracellular vesicles, sEVs originate from the inward budding of multivesicular bodies, and contain a variety of nucleic acids, lipids and proteins derived from their cell-of-origin^12,13^. Upon fusion with the plasma membrane, sEVs are released into the extracellular environment and capable of entering the circulation^10^. It is for this reason that sEV isolation from the body fluids of patients serves as a potential source of novel markers that could characterise NSCLC in more detail compared to currently available clinical techniques.

In this study, we explored the capacity of sEV proteins to act as biomarkers for disease progression in locoregionally confined NSCLC patients. We identified sEV protein changes induced by EMT, in oncogenically transformed lung cells that could be detected in the blood of NSCLC patients. Through a novel sEV protein signature, we were able to stratify disease progression with a sensitivity of 86% and specificity of 96%. This work will have clinical implications as this sEV signature identifies non-metastatic patients that will benefit from adjuvant therapy, providing specific clinical targeting of aggressive tumours without subjecting patients to treatments that would derive no benefit.

## Results

### Generation of an sEV protein signature for NSCLC

**Fig. 1:**
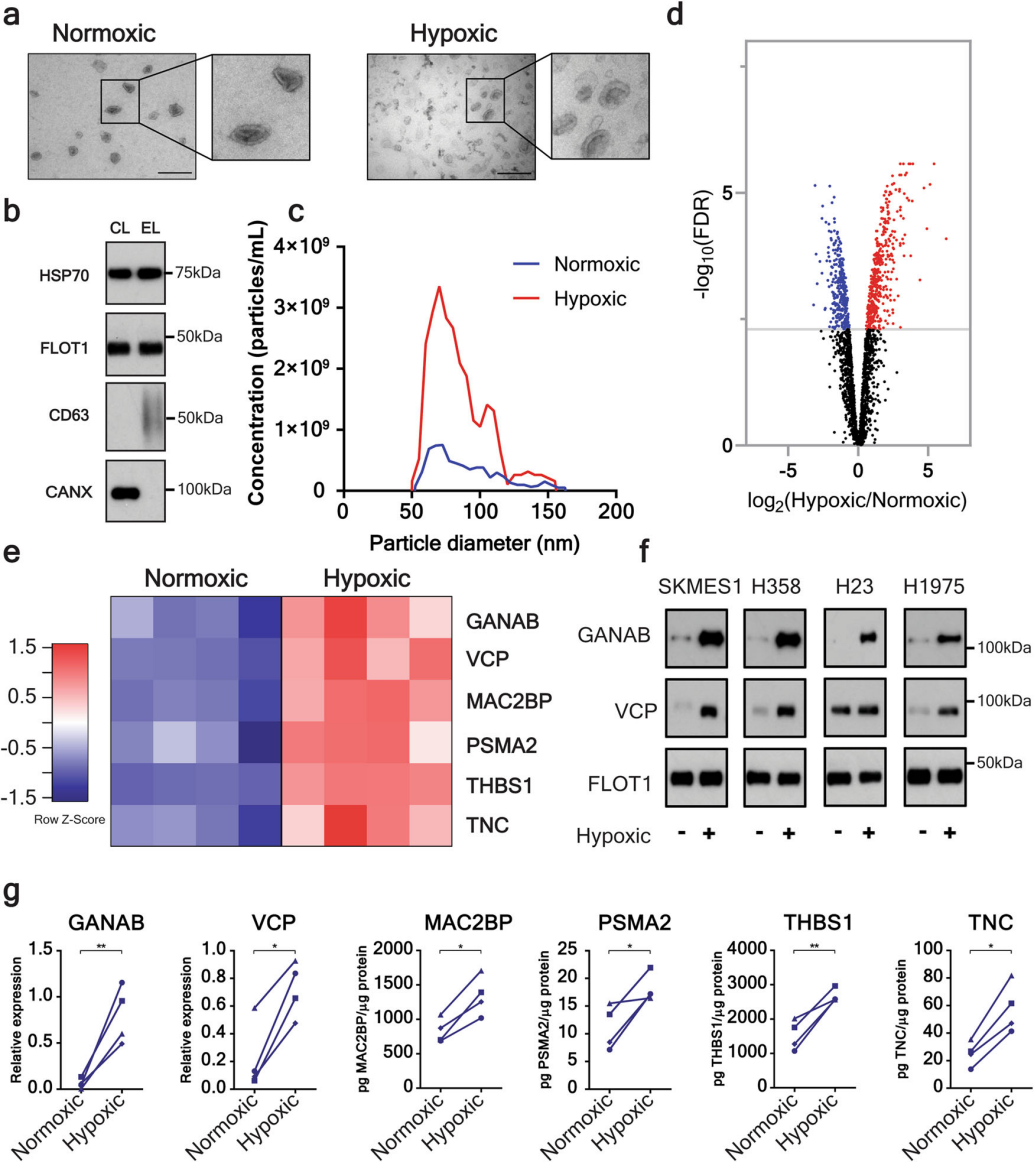
Hypoxia-induced changes to the protein composition of NSCLC cell-derived sEVs. **a** The morphology of isolated sEVs was assessed using transmission electron microscopy. Representative images of normoxic and hypoxic SKMES1-derived sEVs (Size bar 200 nm) also indicate clear upregulation of sEV concentration. **b** Western blot of sEVs from SKMES1 demonstrating the presence of sEV heat shock protein 70 (HSP70), flotillin-1 (FLOT1), and CD63 antigen (CD63) and the absence of the cell marker calnexin (CANX). **c** Nanoparticle analysis using tunable resistive pulse sensing (TRPS) of sEVs isolated from SKMES1 under normoxic and hypoxic conditions demonstrating the majority of sEVs have a size range between 30 and 150 nm, and that hypoxia increases sEV secretion. **d** Volcano plot of quantitative mass spectrometry identifying 426 proteins that are upregulated in SKMES1 sEVs (FDR < 0.005; *n* = 4 independent replicates). **e**, Heatmap demonstrating quantitative mass spectrometry identification of 6 proteins to be significantly upregulated in hypoxic sEVs derived from SKMES1 cells. **f**, **g** Mass spectrometry results were confirmed using western blot of GANAB and VCP (**f**), and ELISA for MAC2BP, PSMA2, THBS1, and TNC in H23, H358, H1975, and SKMES1 NSCLC cell lines (**g**) (▲ – H23, ● – H358, ♦ – H1975, ■ – SKMES1). *p < 0.05, **p < 0.01.

We postulated that NSCLC cells exposed to hypoxic conditions would secrete sEVs with a distinct protein profile, indicative of an aggressive phenotype. We isolated sEVs secreted by human NSCLC lines (H23, H358, H1975, and SKMES1) cultured under normoxic (21% O_2_) or hypoxic (2% O_2_) conditions (Fig. 1a–c; Supplementary Fig. 1). Small EVs displayed typical size distribution and contained canonical sEV markers (Fig. 1a–c; Supplementary Fig. 1a). Transmission electron microscopy (TEM) and nanoparticle analysis revealed NSCLC cells significantly increased sEV secretion in response to hypoxia (Fig. 1a, c; Supplementary Fig. 1b). The proteomes of normoxic and hypoxic sEVs from SKMES1 cells were then evaluated using mass spectrometry. Label-free quantification by spectral intensity identified 736 proteins differentially expressed at an FDR of 0.005 (Fig. 1d; Supplementary Data 1). Of these 736 proteins, 426 were upregulated in hypoxic sEVs derived from SKMES1 cells (Fig. 1d; Supplementary Data 1). Based on the previous association with cancer progression, six upregulated proteins (three cytoplasmic [GANAB^14^, VCP^15^, PSMA2^16^], two extracellular [TNC^17,18^, THBS1^19^], and one transmembrane protein [MAC2BP^20^]) (Fig. 1e) were selected for further investigation in sEVs derived from the adenocarcinoma cell lines H23, H358, and H1975. Western blot and ELISAs of the six biomarkers revealed that all proteins were significantly elevated in hypoxic sEVs derived from the NSCLC cell lines (Fig. 1f, g).

### Assessment of hypoxic sEV signature in other cancers

**Fig. 2:**
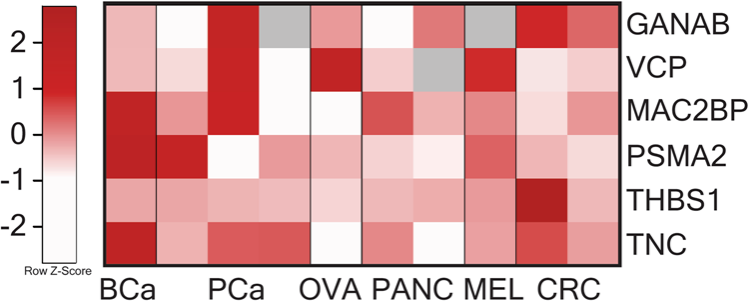
Hypoxic sEV signature is not consistently elevated in other cancers. A total of six different cancer types, including breast cancer (BCa; MDA-MB-231), prostate cancer (PCa; DU145, LNCaP, PC-3), ovarian cancer (OVA; CAOV3), pancreatic cancer (PANC; MIA PaCa-2, PANC1), melanoma (MELA; A375), and colorectal cancer (CRC; HT29, SW620), were investigated for their response to hypoxia. Heatmap representing the hypoxic log_2_ fold change in comparison to sEVs derived from normoxic conditions. Cell lines did not consistently elevate the levels of all 6 proteins, or in some cases the expression of proteins was reduced, particularly evident in OVA and PANC. Grey denotes not detected.

Given the general presence of a hypoxic microenvironment in various solid tumours, we wanted to investigate the general applicability of the hypoxia-induced protein panel. In order to assess this, we evaluated the expression level of these proteins in sEVs derived from cell lines of breast, prostate, ovarian, pancreatic and colorectal cancer as well as melanoma. A total of 10 different cell lines were cultured under normoxic or hypoxic conditions, and sEVs isolated. Upregulation of the hypoxia-induced protein panel observed in NSCLC hypoxic sEVs was not reflected consistently amongst the other cancer types investigated (Fig. 2). Therefore, the hypoxic sEV signature appeared to be cancer-specific, and associated with sEVs derived from NSCLC cells exposed to hypoxic conditions.

### Oncogenic transformation is required for sEV protein changes

**Fig. 3:**
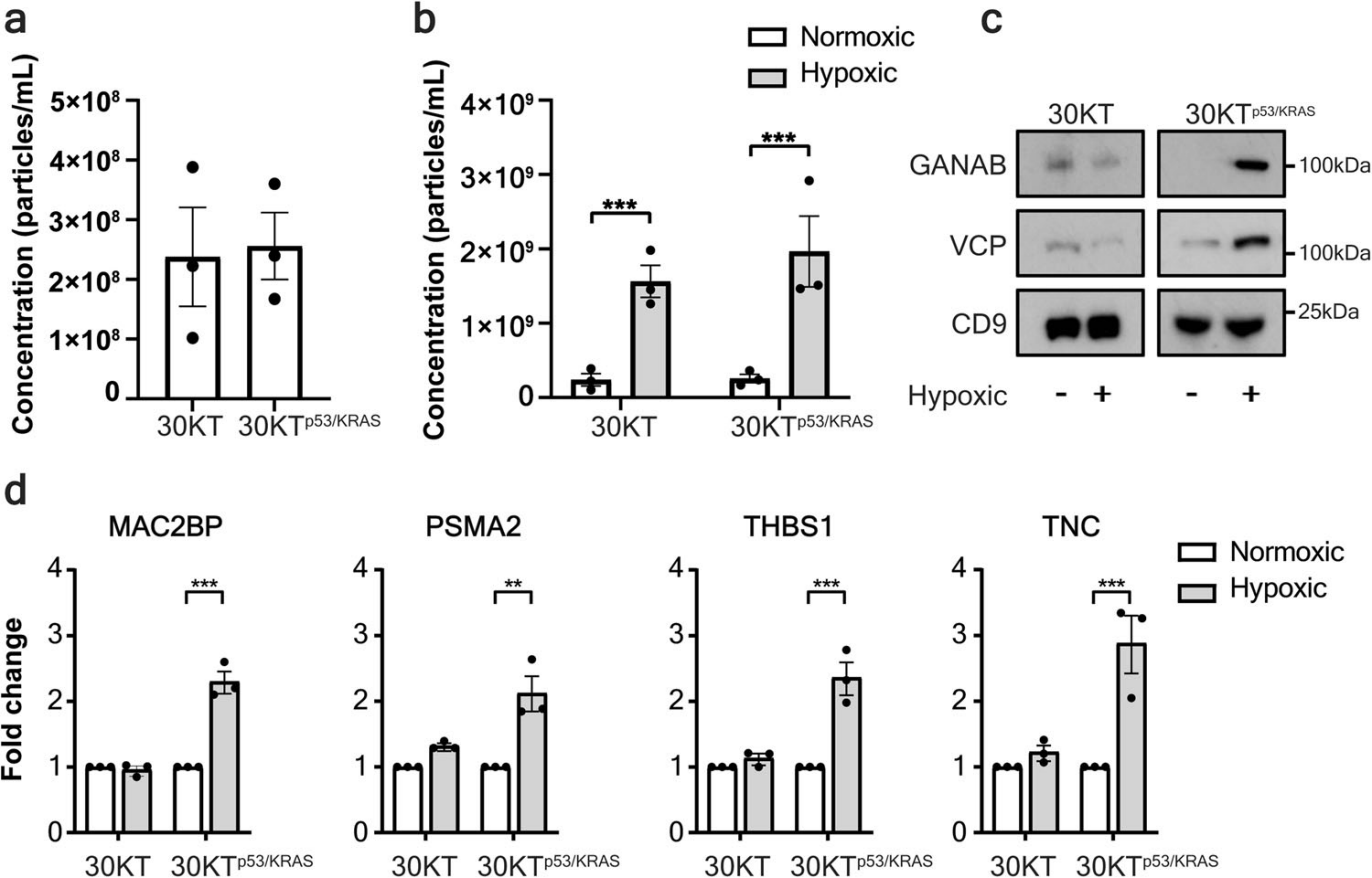
The hypoxic sEV signature is derived from lung cells that are malignantly transformed. **a** Nanoparticle analysis of sEVs isolated from normal or transformed HBECs shows no difference in sEV secretion. **b** Hypoxia significantly increases sEV secretion in HBEC lines independent of the presence of oncogenic manipulations. **c** Western blot of of sEVs from normal lung or malignant HBECs demonstrates that hypoxia elevates the abundance of GANAB and VCP only in malignant HBECs sEVs. **d** ELISA of MAC2BP, PSMA2, THBS1 and TNC in sEVs derived from hypoxic or normoxic conditions of normal or transformed lung epithelial cells indicates that only transformed lung cells increase the abundance of all four proteins in response to hypoxia (±SEM; *n* = 3 independent replicates). **p < 0.01, ***p < 0.001.

To understand the biology of the origin of the hypoxia-induced protein panel, we investigated the potential mechanism underpinning this sEV signature. We hypothesized that transformed lung cells were responsible for elevated levels of the protein signature, given the response to hypoxia is dependent on the cellular context^21^. To determine if the hypoxia-induced protein panel are secreted by normal or transformed lung epithelial cells, we isolated sEVs from a normal, immortalized human bronchial epithelial cell line (HBECs; 30KT) and an isogenic, p53 knockdown and Kras v12 overexpression transformed line (30KT^p53/KRAS^)^22^. Surprisingly, oncogenic transformation alone did not increase sEV secretion (Fig. 3a). However, the increase in sEV secretion observed in NSCLC cell lines in response to hypoxia (Fig. 1c; Supplementary Fig. 1) was also observed from both 30KT and 30KT^p53/KRAS^ cells (Fig. 3b). This indicates that an increase in sEV production is not necessarily associated with a transformed phenotype, but rather a general phenomenon in response to hypoxia, independent of oncogenic context. Although the secretion levels of sEVs remain the same between normal and transformed cells, we hypothesized that the protein cargo of sEVs differs and reflects the parental cell-of-origin. Interestingly, the hypoxia-induced protein panel was not induced by hypoxia in untransformed HBEC-derived sEVs, but was enriched in 30KT^p53/KRAS^ sEVs (Fig. 3c, d). These data indicate that normal lung epithelium does not respond to a hypoxic microenvironment by secreting sEVs enriched for these 6 proteins in vitro, suggesting that this process may be specific to cancerous lesions.

### Evaluation of the sEV protein signature in NSCLC patients

**Fig. 4:**
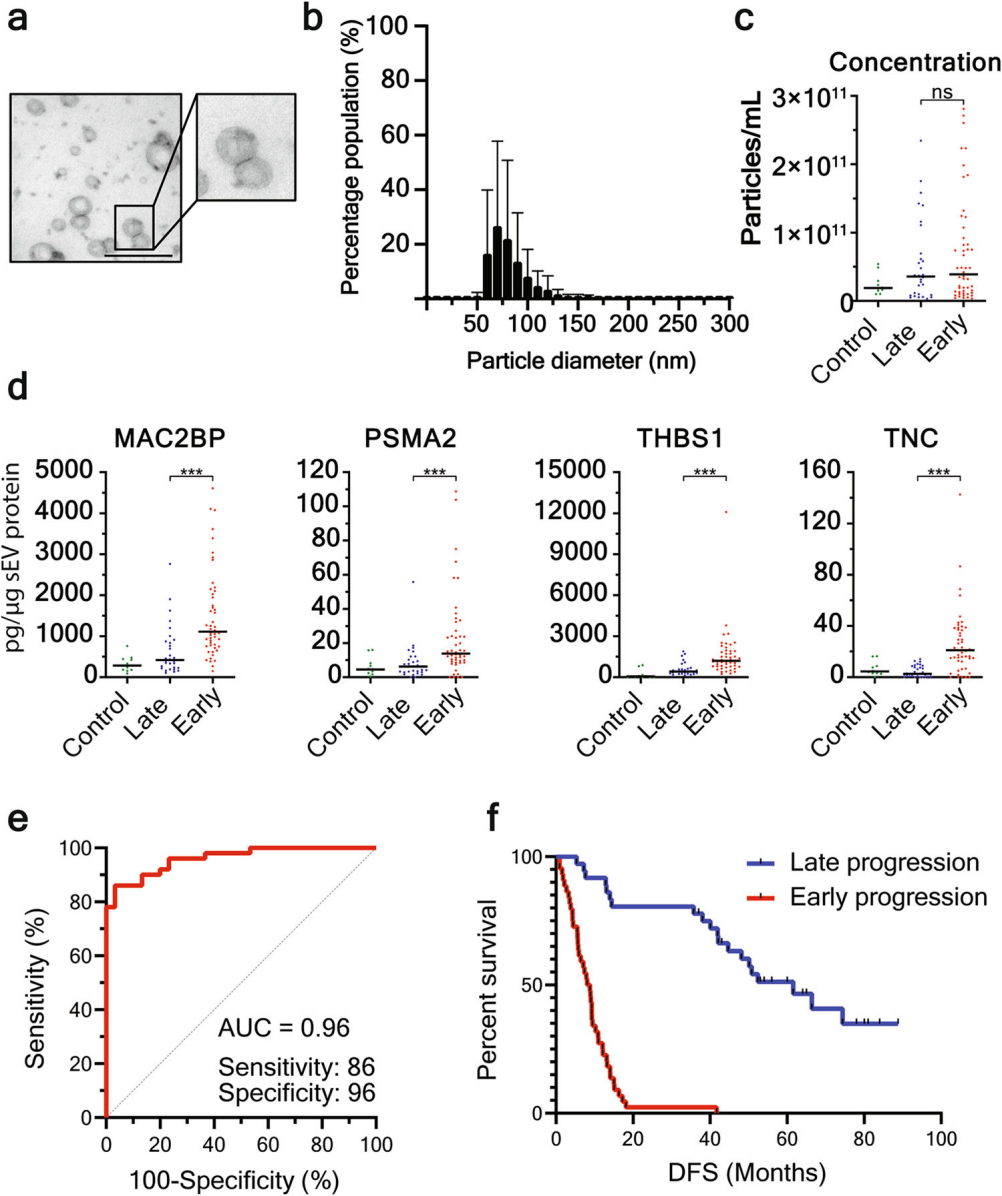
Hypoxic sEV signature prognosticates disease progression in discovery NSCLC patient cohort. **a**, **b** SEVs isolated from NSCLC plasma display typical morphology as shown by TEM (**a**, size bar 200 nm), and size distribution of 30–150 nm (**b**). **c** TRPS demonstrates that there is no difference in sEV concentration in plasma from healthy controls, patients that progress within 18 months (early) or patients without progression at 36 months (late). **d** The hypoxic sEV signature proteins are upregulated in sEVs derived from patients that progress within 18 months. **e** ROC curve demonstrates that the hypoxic sEV signature is an excellent prognostic marker of disease progression (<18 months) in NSCLC patients with a sensitivity of 86% and specificity of 96%. **f** Kaplan–Meier curve shows a clear separation of patient DFS based on the abundance of proteins from the hypoxic sEV signature. ***p < 0.001.

We next postulated that hypoxic-induced sEV changes could be utilised as a prognostic biomarker for disease progression in locoregionally confined NSCLC. Typical end point clinical trials are based on overall survival, and progression is a less precise variable that is related to the frequency and type of follow-up^23^. In particular, 18 months progression-free survival was selected as the separation between early and late progression as most follow-up protocols change in frequency and nature 12 months after the end of therapy. Small EVs were isolated from the plasma of an 80-patient, stage I–III NSCLC discovery cohort (Fig. 4a, b; Supplementary Table 1). Although hypoxia increases sEV secretion from NSCLC cells (Fig. 1c; 3b; Supplementary Fig. 1), we found that sEV concentration in the plasma of NSCLC patients had no prognostic value for early clinical progression (within 18 months), nor was there a difference of plasma sEVs between healthy and NSCLC patients (*p* > 0.05) (Fig. 4c). This might be related to the earlier observation that oncogenic mutation or hypoxic conditions resulted in no difference in sEV secretion compared to normal cells (Fig. 3a, b) as well as that cancer-derived sEVs likely are only a small constituent of all blood sEVs. In order to achieve quantitative and sensitive detection, we investigated four of the hypoxic sEV proteins by ELISA (MAC2BP, PSMA2, THBS1, TNC). Interestingly, these four proteins were increased significantly in sEVs derived from NSCLC subjects who experienced early disease progression (Fig. 4d). Each protein from the hypoxic signature was, to an extent, individually prognostic for disease progression, as assessed by receiver operating characteristic (ROC) curves (Supplementary Fig. 2). However, by combining the four proteins through logistic regression analysis, we were able to clearly differentiate NSCLC patients based on the abundance of these 4 sEV proteins, with an AUC of 0.96 (Youden’s index, sensitivity 86%; specificity 96%; Fig. 4e). Moreover, this approach also generated a highly significant separation classification of NSCLC patient disease-free survival (DFS) that could be used to provide clinical guidance on the likelihood of disease progression (Fig. 4f).

### EMT of NSCLC cells is causative for hypoxic sEV protein signature

**Fig. 5:**
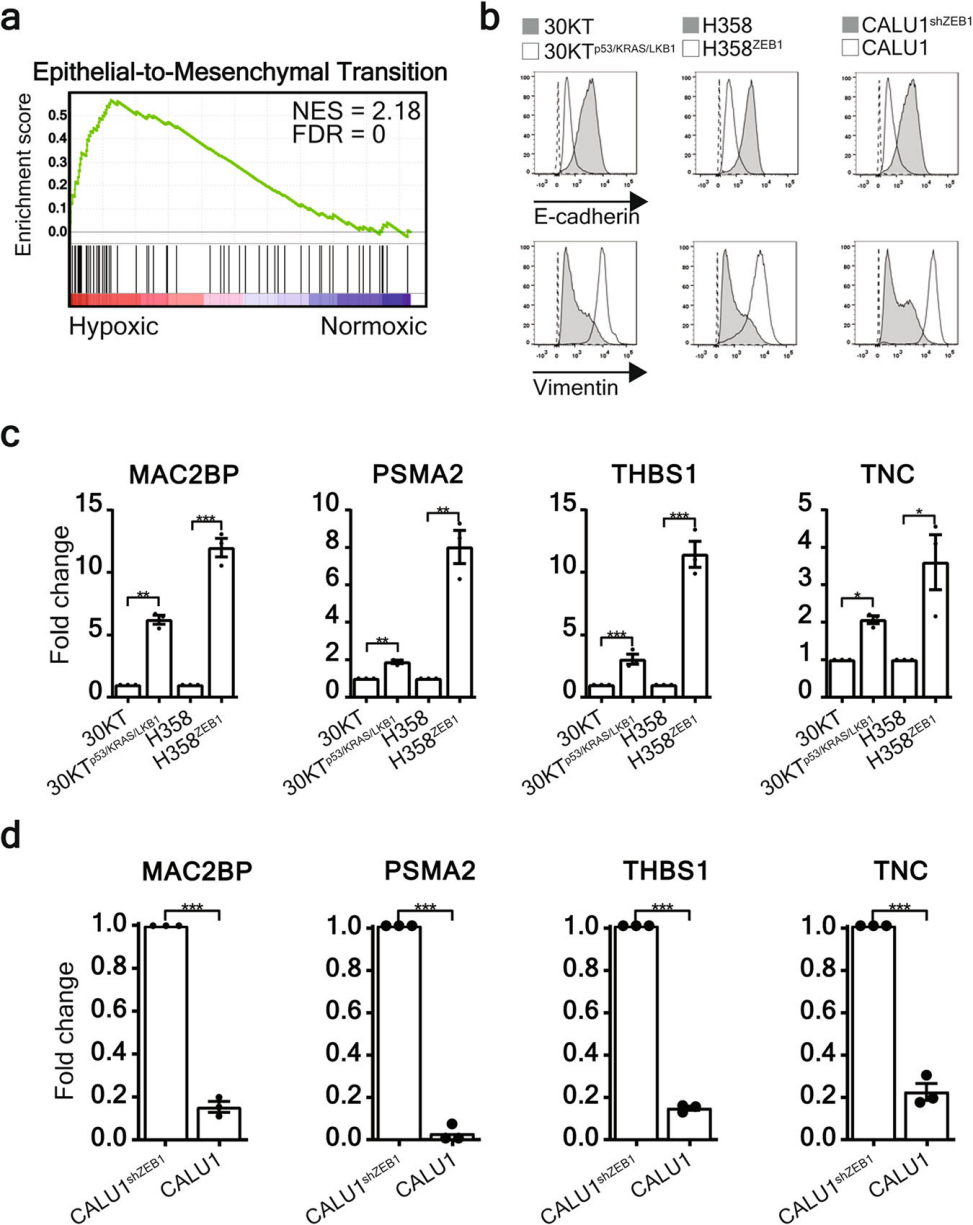
The hypoxic sEV signature is derived from lung cells that have undergone EMT. **a** GSEA identified the hallmark epithelial-to-mesenchymal transition gene set was significantly associated with sEVs derived from hypoxic SKMES1 cells. **b** Oncogenically induced EMT in mesenchymal 30KT^p53/KRAS/LKB1^ and H358^ZEB1^ cells was demonstrated by loss of E-cadherin and gain of vimentin expression assessed by flow cytometry, whereas MET was induced in CALU1 cells through knockdown of ZEB1 (CALU1^shZEB1^) resulting in the gain of E-cadherin and loss of vimentin expression. **c** ELISA of MAC2BP, PSMA2, THBS1 and TNC demonstrates that EMT induces an increase of all four signature proteins in sEVs (±SEM; *n* = 3). **d** Conversely, when MET is induced in CALU1 cells the abundance of all four signature proteins is significantly reduced (±SEM; *n* = 3 independent replicates). *p < 0.05, **p < 0.01 ***p < 0.001.

We previously demonstrated that the protein content of sEVs reflects the phenotype of the cell-of-origin^13,24^. To further examine the phenotype of cells that secrete elevated levels of the hypoxic sEV signature, we performed gene set enrichment analysis (GSEA) using protein abundance estimates in sEVs derived from normoxic or hypoxic SKMES1 cells. Interestingly, among a number of gene sets (Supplementary Fig. 3), the top ranked gene set enriched in hypoxic SKMES1 sEVs was associated with EMT (Fig. 5a). Given that hypoxia is a strong inducer of EMT in cancer cells^25^, we postulated that a mesenchymal phenotype alone could be sufficient to cause sEV protein signature elevation. Oncogenically induced EMT in the 30KT line through p53 knockdown, Kras v12 overexpression and LKB1 knockdown (30KT^p53/KRAS/LKB1^)^26^, as well as ZEB1 overexpression in H358 (H358^ZEB1^) cells resulted in reduced E-cadherin, and increased expression of the mesenchymal marker vimentin (Fig. 5b). In contrast, knockdown of ZEB1 in mesenchymal CALU1 cells (CALU1^shZEB1^) subsequently caused mesenchymal-to-epithelial transition (MET) by increasing E-cadherin and reducing vimentin expression (Fig. 5b). Strikingly, oncogenically induced EMT resulted in elevated sEV hypoxic signature proteins under normoxic conditions (Fig. 5c). Conversely, MET of CALU1 cells significantly reduced the expression of all signature proteins (Fig. 5d), indicating the signature proteins are enriched in sEVs derived from mesenchymal lung cancer cells and as a result can identify cancer cells that are highly metastatic and therapy-resistant^26–28^.

### Validation of the sEV signature in an independent NSCLC cohort

**Fig. 6:**
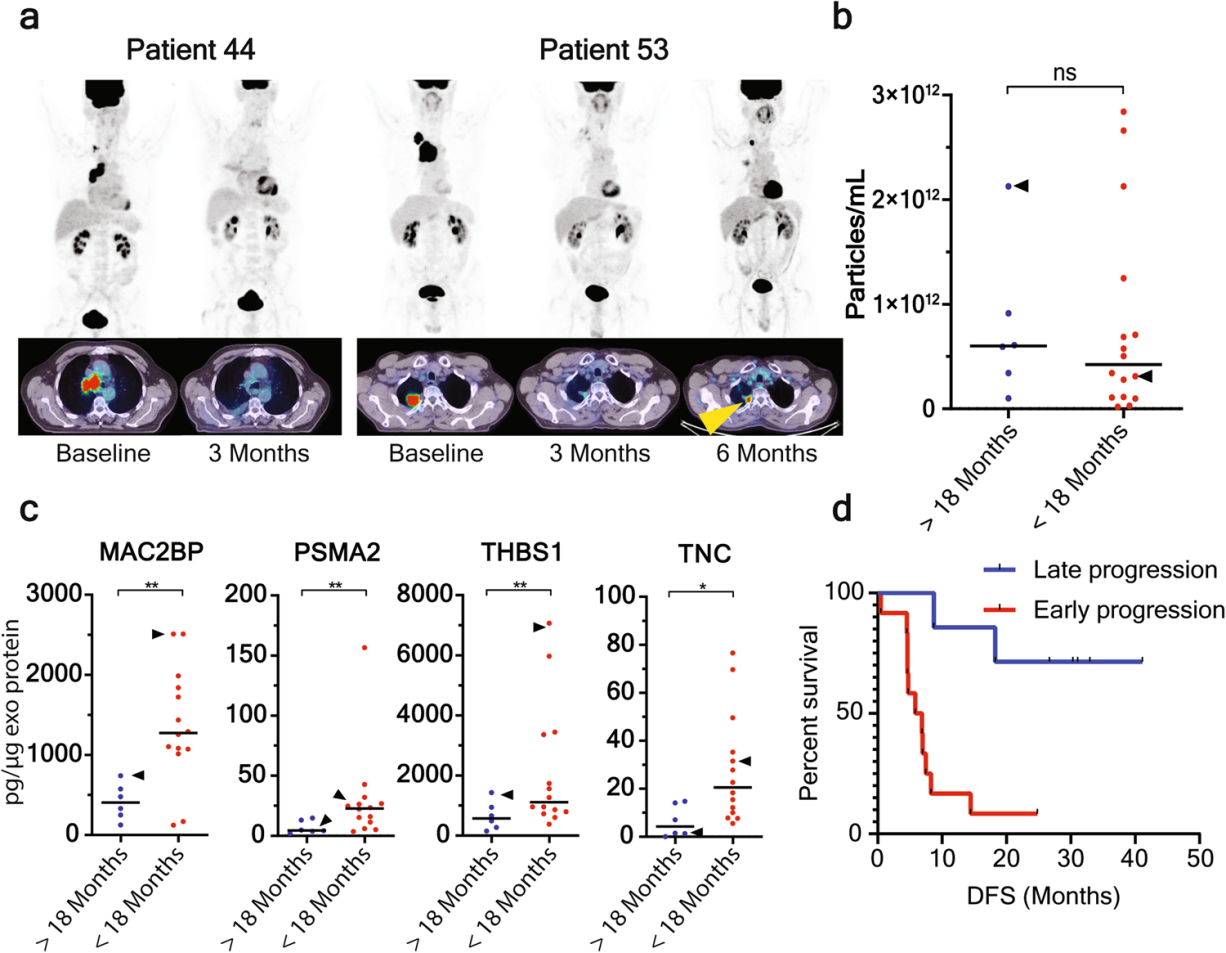
Confirmation that the hypoxic sEV signature prognosticates disease progression in NSCLC patients. **a** ^18^F-FDG PET/CT images of two patients (patient 44 and 53 of the confirmation cohort) that are tracked in *B* and *C* at indicated points (yellow arrowhead; site of progression). **b** In support of the discovery cohort, there was no significant difference in sEV concentration in patients that progress within 18 months compared to patients that progress after 18 months. Patient 44 and 53 are indicated by arrowheads. **c** The four signature proteins are significantly elevated in sEVs derived from patients that progress within 18 months compared to patients without progress at 18 months. **d** Kaplan–Meier plot of DFS of NSCLC patients using the logistic model from the discovery cohort indicates a clear separation in DFS. *p < 0.05, **p < 0.01.

For independent validation of our clinical data, we evaluated the sEV content of 20 locoregionally advanced NSCLC subjects treated with curative-intent chemoradiation, sampled at baseline and longitudinally monitored with 18F-FDG and 18F-FLT PET/CT (Fig. 6a; Supplementary Table 2). We expected that patients with a high abundance of signature proteins would be treatment-refractory and have short DFS. In agreement with the discovery cohort findings, there was no difference in sEV concentration (Fig. 6b), however, the hypoxic/EMT sEV signature showed significant elevation and prognostic value in subjects who progressed within 18 months, compared to those who did not progress within 18 months (Fig. 6c, d). Using the logistic model established in the discovery cohort, the signature was capable of separating patients who experienced disease progression within 18 months and patients that progressed after 18 months (Fig. 6d).

## Discussion

Lung cancer remains the major cause of cancer deaths and despite curative-intent treatment, a large proportion of patients experience progressive disease. Metastatic disease is responsible for over 90% of all cancer-related deaths^29^. It is well established that hypoxia occurs during early tumour development and causes an aggressive, treatment-resistant, invasive and metastatic phenotype^5,21,30^. Within the tumour microenvironment, hypoxia alters numerous physiological functions, including angiogenesis, metabolism, immune cell function and EMT, thereby promoting metastasis^31^. EMT involves the phenotypic depolarisation of epithelial cells into elongated mesenchymal cells and is a pivotal process in the metastatic cascade^6^. Importantly, partial EMT, which result in a hybrid EMT state, has been shown to be clinically relevant in many cancers^32^. The functional consequence of EMT or partial EMT in cancer includes enhanced cell invasion, migration, altered tumour microenvironment immune cell composition^8^ and resistance to apoptotic stimuli, such as chemotherapy^6,9^. The association of EMT with aggressive, therapy-refractive tumours has been verified in several cancer types^9,25,33^. In lung cancer, loss of E-cadherin expression (an early canonical step in EMT), and co-expression of hypoxia-inducible factor 1α (HIF1α) and EMT transcription factors Twist and Snail1, is associated with a therapy-refractive and metastatic phenotype^34^. Moreover, EMT transcription factors can be detected in NSCLC patient tumours at a potentially curable stage, and correlate with survival^35^. Understanding the mechanisms that drive tumour metastasis, and how hypoxia/EMT contributes to the process, is key in developing effective therapies that can reduce the high mortality rate.

Currently, pathology-based TNM staging is the most important factor determining the likelihood of relapse or progression. However, it is currently impossible to stratify patients into responding and non-responding categories with sufficient accuracy. Prognostic biomarkers could yield important clinical information, allowing clinicians to determine which patients may benefit from adjuvant chemotherapy, and those who will suffer from adverse related events with no benefit. A robust, blood-based biomarker panel capable of identifying patients with aggressive and resistant disease will pave the way for optimising and improving adjuvant treatments after first line surgery and chemoradiation treatments, in a truly precision-medicine and personalized management approach.

The possibility of accurately predicting clinical outcome based on a simple blood test has immense implications for personalized patient management and triaging. Liquid biomarkers provide an attractive alternative in the prognosis of NSCLC patients as they are minimally invasive. There has been much work conducted on blood-contained markers for diagnostic and prognostic/predictive purposes. Early work assessing mass spectrometry-based analysis of cancer-derived protein markers did not yield a reliable clinical tool for diagnostic and/or prognostic purposes for NSCLC, due to the dynamic range issues that limit biomarker identification in the plasma proteome^36^. Recently, liquid biopsies have focused on circulating DNA and circulating tumours cells. However, both approaches are technically challenging, and have yet to provide sufficient sensitivity and specificity in the prognostication of NSCLC patients and prediction of treatment response.

Circulating sEVs have been shown to have excellent diagnostic and prognostic value in various cancers^37–39^. We now expand on the utility of sEVs in patient management by identifying an sEV signature that is highly effective in the prognosis of NSCLC patient progression. Our work is consistent with previously published reports on the role of EMT in the progression of cancers, including NSCLC^9,33,35^. Given that EMT is known to cause a pro-metastatic and chemoresistant phenotype, our sEV signature has the potential to identify patients at the earliest stage that would benefit from the intensification of treatment or adjuvant treatment interventions. Moreover, we have previously demonstrated that EMT-induced chemoresistance and metastatic potential can be transferred into recipient lung cancer cells via sEVs^26^. Therefore, sEV identification of EMT in the primary tumour can identify aggressive tumours as well as provide insight into the promotion of metastatic and chemoresistant phenotypes.

The major barriers of developing prognostic (and ultimately predictive) markers for lung cancer are the lack of reliable biomarkers with the desired specificity and sensitivity, as well as the capability of detecting these biomarkers in an inexpensive and rapidly deployable technique. This sEV signature has the potential to complement TNM staging, to provide a biomarker for therapy monitoring and to detect disease relapse, and to allow for specific tailoring of treatment interventions and ultimately improve clinical outcome.

## Methods

### Cell culture

Cell line authentication was carried out using short tandem repeat profiling. All cell lines were repeatedly tested negative for mycoplasma in house at QIMR Berghofer. Human NSCLC cell lines H23, H358, H1975, and SKMES1; breast cancer MDA-MB-231; prostate cancer DU145, LNCaP, and PC-3; ovarian cancer CAOV3; pancreatic cancer MIA PaCa-2, PANC1; melanoma A375; and colorectal cancer HT29, SW620 were obtained from ATCC. Isogenic immortalized normal human bronchial epithelial cells (HBEC30KT) transformed with p53 knockdown (30KT^p53^), p53 knockdown and Kras v12 overexpression (30KT^p53/KRAS^) and p53 knockdown, Kras v12 overexpression, and LKB1 knockdown (30KT^p53/KRAS/LKB1^)^22^, H358-pMSCV (H358) and H358-pMSCV-ZEB1 (H358^ZEB1^), CALU1-pSRP (CALU1^pSRP^), and CALU1-pSRP-shZEB1 (CALU1^shZEB1^) were a gift from Dr. Jill Larsen^35,40^. Cancer cell lines were maintained in DMEM or RPMI supplemented with 10% foetal bovine serum, 100 U/mL penicillin and 100 mg/mL streptomycin and incubated at 37 °C in 5% CO_2_. HBECs were cultured in keratinocyte serum free medium (KSFM), supplemented with EGF (5 ng/ mL) and bovine pituitary extract (50 mg/L), 37 °C in 5% CO_2_. Cell conditioned media (CCM) from NSCLC cell lines were collected from cells cultured under normoxic (21% O_2_) or hypoxic (2% O_2_) conditions in serum-free media. CCM was collected from HBEC cells conditioned under normoxic or hypoxic conditions in KSFM depleted of bovine sEVs through overnight centrifugation at 100,000 *g*_avg_.

### Antibodies and reagents

The following antibodies were used for Western blotting: Calnexin (1:1000; Cell Signalling Technology, 2679 S), CD9 (1:2000; Abcam, ab92726), CD63 (1:1000; Abcam, ab8219), Flotillin-1 (1:1000; BD Transduction Laboratories, 610821), HSP70 (1:1000; Transduction Laboratories, 610608), TSG101 (1:500; Santa Cruz, sc-6037). Horseradish peroxidase (HRP)-conjugated secondary antibodies were purchased from Thermo Scientific. MAC2BP, PSMA2, and THBS1 ELISA DuoSets were purchased from R & D Systems, TNC ELISA kits were purchased from Abcam. qEV columns were purchased from Izon and stored in PBS (0.1% sodium azide) at 4 °C. OptiPrep^TM^ was purchased from Sigma-Aldrich.

### Western blot analysis

Western blots were performed as previously described^26,41^. Briefly, proteins were resolved by SDS-PAGE, transferred to polyvinylidene fluoride membranes, blocked in 5% non-fat powdered milk in PBS-T (0.5% Tween-20) and probed with antibodies. Protein bands were detected with enhanced chemiluminescence reagent (Amersham ECL Select). Protein bands were quantified with ImageJ and normalized to a loading control.

### sEV isolation and analysis

sEVs were isolated and analysed as previously described^41,42^. For sEV isolations from in vitro cell culture, CCM was centrifuged at 300 *g* for 10 min at 4 °C and filtered through 0.22 µm filters to remove floating cells and large extracellular vesicles. Clarified CCM was then concentrated to 500 µL and overlaid on a discontinuous iodixanol density gradient and centrifuged for 16 h at 100,000 *g*_avg_ at 4 °C. SEV containing fractions were diluted to 20 mL in PBS and centrifuged at 100,000 *g*_avg_ at 4 °C for 2 h. The resulting pellet was resuspended in PBS and stored at −80 °C until use. For the isolation of sEVs from human plasma, plasma was thawed at room temperature and prepared by removing remaining platelets and large vesicles by centrifugation at 1500 *g* and 10,000 *g*, for 10 and 20 min respectively. Prepared plasma was overlaid on a size exclusion column followed by elution with PBS, concentrated in Amicon® Ultra-4 10 kDa nominal molecular weight centrifugal filter units and stored at −80 °C until use. SEV isolations from cell culture and human plasma were confirmed with western blot, tunable resistive pulse sensing (TRPS), and transmission electron microscopy as previously described^41^.

### Mass spectrometry

LCMS analysis of sEV digests was performed by interfacing a NanoAcquity UPLC (Waters) in front of an Elite Orbitrap ETD mass spectrometer (Thermo Fisher Scientific). Protein identification and label-free quantification were performed using MaxQuant (version 1.5.7.4^43^) as previously described^44^. A false discovery rate (FDR) threshold of 0.01 was used for peptide and protein identifications.

### Patient cohorts

This study was approved for the collection and use of all clinical samples by the human research ethics committees of The Prince Charles Hospital (HREC/16/QPCH/281), The University of Queensland (2017001730), the Peter MacCallum Cancer Centre (2008001483) and the QIMR Berghofer (P2180). Informed consent was received from all patients. Retrospective analysis of a discovery cohort of 80 patient plasma samples, collected between 2001–2015 sampled at baseline or shortly after surgery (≤6 days [*n* = 17]) was conducted. Stage I–IIIA patients received front line surgery with curative intent and were separated into two categories based on disease recurrence before 18 months (early progression), or no recurrence within 18 months (late progression) of complete resection.

The independent confirmation cohort samples were collected as part of a trial at the Peter MacCallum Cancer Centre (ACTRN12611001283965) and included 20 patients who provided informed consent to participate in an ERB approved prospective trial of sequential ^18^F-FDG PET/CT and ^18^F-Fluorothymidine (FLT) PET/CT imaging prior to, during and after curative intent chemo- radiotherapy (RT)^45^. As previously reported, eligibility for this trial included a staging ^18^F-FDG PET/CT scan, histological or cytological confirmation of stage I–III NSCLC, with an Eastern Cooperative Oncology Group (ECOG) performance status of 0–1^46^. Exclusion criteria included previous thoracic radiotherapy and complete surgical tumour excision. Patients received concurrent chemoradiation in accordance with two standardised protocols. RT consisted of 60 Gy in 30 fractions over 6 weeks. One of two chemotherapy regimens was administered: either weekly carboplatin [area under curve, two intravenously] and paclitaxel [45 mg/m^2^ intravenously] for older patients or those with significant comorbidities; or cisplatin [50 mg/m^2^ intravenously] on days 1, 8, 29, and 36 and etoposide [50 mg/m^2^ intravenously] during weeks 1 and 5 for younger fitter patients. ^18^F-FDG PET/CT scans were acquired at baseline, Day 10, Day 24 and Day 90. Ongoing monitoring was performed with standard CT imaging at 3 monthly intervals for 12 months and 6-monthly intervals thereafter.

### Gene set enrichment analysis

Gene set enrichment analysis (GSEA)^47^, version 2.2.3, was used to identify enriched pathways in sEVs isolated from hypoxic SKMES1 cells as previously described^24^. Protein intensity values of all proteins in sEVs derived from normoxic or hypoxic SKMES1 sEVs were analysed using the Molecular Signatures Database (MSigDB). Analysis was performed using the Hallmark gene sets database (version 5.2), Signal2Noise ranking metric, 1000 phenotype permutations, and a weighted enrichment statistic.

### Statistics and reproducibility

GraphPad Prism version 6.0, EdgeR version 2.6.10, MedCalc version 16.8.4, and IPA were used for all calculations. Unpaired Student’s t-test was used to calculate the difference in expression values of proteins from sEVs in vitro. Subcellular localisation of proteins was generated through IPA (QIAGEN Inc). Multiple comparisons were controlled for using the Sidak-Bonferroni method for assessing proteins in patient-derived sEVs. A negative-binomial exact test was used to assess the mass spectrometry derived spectral counts, where the Benjamini-Hochberg adjustment was applied to control the FDR. Univariate analysis using the log-rank test was used to assess disease-free survival (Kaplan–Meier curves). All in vitro experiments had a minimum of three independent replicates. Differences with *p*-values < 0.05 were considered significant (*p < 0.05, **p < 0.01, ***p < 0.001), with the exception of a FDR threshold of 0.001.

### Reporting summary

Further information on research design is available in the Nature Portfolio Reporting Summary linked to this article.

## Supplementary information

Supplementary Information

Description of Additional Supplementary Files

Supplementary Data 1

Reporting Summary

## Data Availability

All data generated or analysed during this study are included in this published article (and its supplementary information files). Full length western blots are available in Supplementary Fig. 4. Source data has been uploaded to Figshare (10.6084/m9.figshare.21405510). The mass spectrometry proteomics data have been deposited to the ProteomeXchange Consortium via the PRIDE^48^ partner repository with the dataset identifier PXD038587.

## References

[1] Torre LA (2015). Global cancer statistics, 2012. CA: Cancer J. Clin..

[2] Sung H (2021). Global cancer statistics 2020: GLOBOCAN estimates of incidence and mortality worldwide for 36 cancers in 185 countries. CA Cancer J. Clin..

[3] Molina JR, Yang P, Cassivi SD, Schild SE, Adjei AA (2008). Non-small cell lung cancer: epidemiology, risk factors, treatment, and survivorship. Mayo Clin. Proc..

[4] Zhu T (2020). Mechanisms and future of non-small cell lung cancer metastasis. Front Oncol..

[5] Vaupel P, Mayer A (2007). Hypoxia in cancer: significance and impact on clinical outcome. Cancer Metastasis Rev..

[6] Kalluri R, Weinberg RA (2009). The basics of epithelial-mesenchymal transition. J. Clin. Investig..

[7] Yang J (2020). Guidelines and definitions for research on epithelial-mesenchymal transition. Nat. Rev. Mol. Cell Biol..

[8] Lou Y (2016). Epithelial-mesenchymal transition is associated with a distinct tumor microenvironment including elevation of inflammatory signals and multiple immune checkpoints in lung adenocarcinoma. Clin. Cancer Res..

[9] Fischer KR (2015). Epithelial-to-mesenchymal transition is not required for lung metastasis but contributes to chemoresistance. Nature.

[10] Moller A, Lobb RJ (2020). The evolving translational potential of small extracellular vesicles in cancer. Nat. Rev. Cancer.

[11] Kalluri, R. & LeBleu V. S. The biology, function, and biomedical applications of exosomes. *Science* **367**, eaau6977 (2020). 10.1126/science.aau6977 PMC7717626 32029601

[12] Lobb, R. J., Lima, L. G. & Moller A. Exosomes: Key mediators of metastasis and pre-metastatic niche formation. *Seminars Cell Dev. Biol.* **67**, 3–10 (2017). 10.1016/j.semcdb.2017.01.004 28077297

[13] Wen SW (2019). Breast cancer-derived exosomes reflect the cell-of-origin phenotype. Proteomics.

[14] Qin Y (2017). MeCP2 regulated glycogenes contribute to proliferation and apoptosis of gastric cancer cells. Glycobiology.

[15] Peng J (2013). VCP gene variation predicts outcome of advanced non-small-cell lung cancer platinum-based chemotherapy. Tumour Biol..

[16] Denlinger CE, Rundall BK, Keller MD, Jones DR (2004). Proteasome inhibition sensitizes non-small-cell lung cancer to gemcitabine-induced apoptosis. Ann. Thorac. Surg..

[17] Tang YA (2015). Global Oct4 target gene analysis reveals novel downstream PTEN and TNC genes required for drug-resistance and metastasis in lung cancer. Nucleic Acids Res..

[18] Oskarsson T (2011). Breast cancer cells produce tenascin C as a metastatic niche component to colonize the lungs. Nat. Med..

[19] Kudo-Saito C, Shirako H, Takeuchi T, Kawakami Y (2009). Cancer metastasis is accelerated through immunosuppression during Snail-induced EMT of cancer cells. Cancer Cell.

[20] Marchetti A (2002). Expression of 90K (Mac-2 BP) correlates with distant metastasis and predicts survival in stage I non-small cell lung cancer patients. Cancer Res..

[21] Hockel M, Vaupel P (2001). Tumor hypoxia: definitions and current clinical, biologic, and molecular aspects. J. Natl Cancer Inst..

[22] Kim HS (2013). Systematic identification of molecular subtype-selective vulnerabilities in non-small-cell lung cancer. Cell.

[23] Pilz LR, Manegold C, Schmid-Bindert G (2012). Statistical considerations and endpoints for clinical lung cancer studies: Can progression free survival (PFS) substitute overall survival (OS) as a valid endpoint in clinical trials for advanced non-small-cell lung cancer?. Transl. Lung Cancer Res..

[24] Lobb R. J. et al. Oncogenic transformation of lung cells results in distinct exosome protein profile similar to the cell of origin. *Proteomics*10.1002/pmic.201600432 (2017). 10.1002/pmic.201600432 28722786

[25] Thiery JP, Acloque H, Huang RY, Nieto MA (2009). Epithelial-mesenchymal transitions in development and disease. Cell.

[26] Lobb RJ (2017). Exosomes derived from mesenchymal non-small cell lung cancer cells promote chemoresistance. Int J. Cancer.

[27] Stemmler MP, Eccles RL, Brabletz S, Brabletz T (2019). Non-redundant functions of EMT transcription factors. Nat. Cell Biol..

[28] Brabletz T (2012). To differentiate or not-routes towards metastasis. Nat. Rev. Cancer.

[29] Gupta GP, Massague J (2006). Cancer metastasis: building a framework. Cell.

[30] Salem A. et al. Targeting hypoxia to improve non-small cell lung cancer outcome. *J. Natl Cancer Inst*. **110**, 14–30 (2018). 10.1093/jnci/djx160 28922791

[31] Scharping NE, Menk AV, Whetstone RD, Zeng X, Delgoffe GM (2017). Efficacy of PD-1 blockade is potentiated by metformin-induced reduction of tumor hypoxia. Cancer Immunol. Res.

[32] Jolly MK (2022). Measuring and modelling the epithelial- mesenchymal hybrid state in cancer: clinical implications. Cells Tissues Organs.

[33] Zheng X (2015). Epithelial-to-mesenchymal transition is dispensable for metastasis but induces chemoresistance in pancreatic cancer. Nature.

[34] Xiao D, He J (2010). Epithelial mesenchymal transition and lung cancer. J. Thorac. Dis..

[35] Larsen JE (2016). ZEB1 drives epithelial-to-mesenchymal transition in lung cancer. J. Clin. Investig..

[36] Hortin GL, Sviridov D (2010). The dynamic range problem in the analysis of the plasma proteome. J. Proteom..

[37] Hoshino A (2015). Tumour exosome integrins determine organotropic metastasis. Nature.

[38] Melo SA (2015). Glypican-1 identifies cancer exosomes and detects early pancreatic cancer. Nature.

[39] Hoshino A (2020). Extracellular vesicle and particle biomarkers define multiple human cancers. Cell.

[40] Sato M (2006). Multiple oncogenic changes (K-RAS(V12), p53 knockdown, mutant EGFRs, p16 bypass, telomerase) are not sufficient to confer a full malignant phenotype on human bronchial epithelial cells. Cancer Res..

[41] Lobb RJ (2015). Optimized exosome isolation protocol for cell culture supernatant and human plasma. J. Extracell. Vesicles.

[42] Lobb R, Moller A (2017). Size exclusion chromatography: a simple and reliable method for exosome purification. Methods Mol. Biol..

[43] Cox J, Mann M (2008). MaxQuant enables high peptide identification rates, individualized p.p.b.-range mass accuracies and proteome-wide protein quantification. Nat. Biotechnol..

[44] Lobb R. J. et al. Oncogenic transformation of lung cells results in distinct exosome protein profile similar to the cell of origin. *Proteomics*10.1002/pmic.201600432 (2017). 10.1002/pmic.201600432 28722786

[45] Everitt S (2017). Prospective study of serial imaging comparing fluorodeoxyglucose Positron Emission Tomography (PET) and fluorothymidine PET during radical chemoradiation for non-small cell lung cancer: reduction of detectable proliferation associated with worse survival. Int J. Radiat. Oncol. Biol. Phys..

[46] Everitt SJ (2014). Differential (18)F-FDG and (18)F-FLT uptake on serial PET/CT imaging before and during definitive chemoradiation for non-small cell lung cancer. J. Nucl. Med..

[47] Subramanian A (2005). Gene set enrichment analysis: a knowledge-based approach for interpreting genome-wide expression profiles. Proc. Natl Acad. Sci. USA.

[48] Perez-Riverol Y (2022). The PRIDE database resources in 2022: a hub for mass spectrometry-based proteomics evidences. Nucleic Acids Res..

