# Supplementary material for: An epithelial-to-mesenchymal transition induced extracellular vesicle prognostic signature in non-small cell lung cancer
Source: Commun Biol. 2023 Jan 18;6:68. doi: 10.1038/s42003-022-04350-4 (PMC9849257; doi:10.1038/s42003-022-04350-4)
Supplement: Supplementary file 1 — Supplementary Information [file 42003_2022_4350_MOESM1_ESM.pdf]

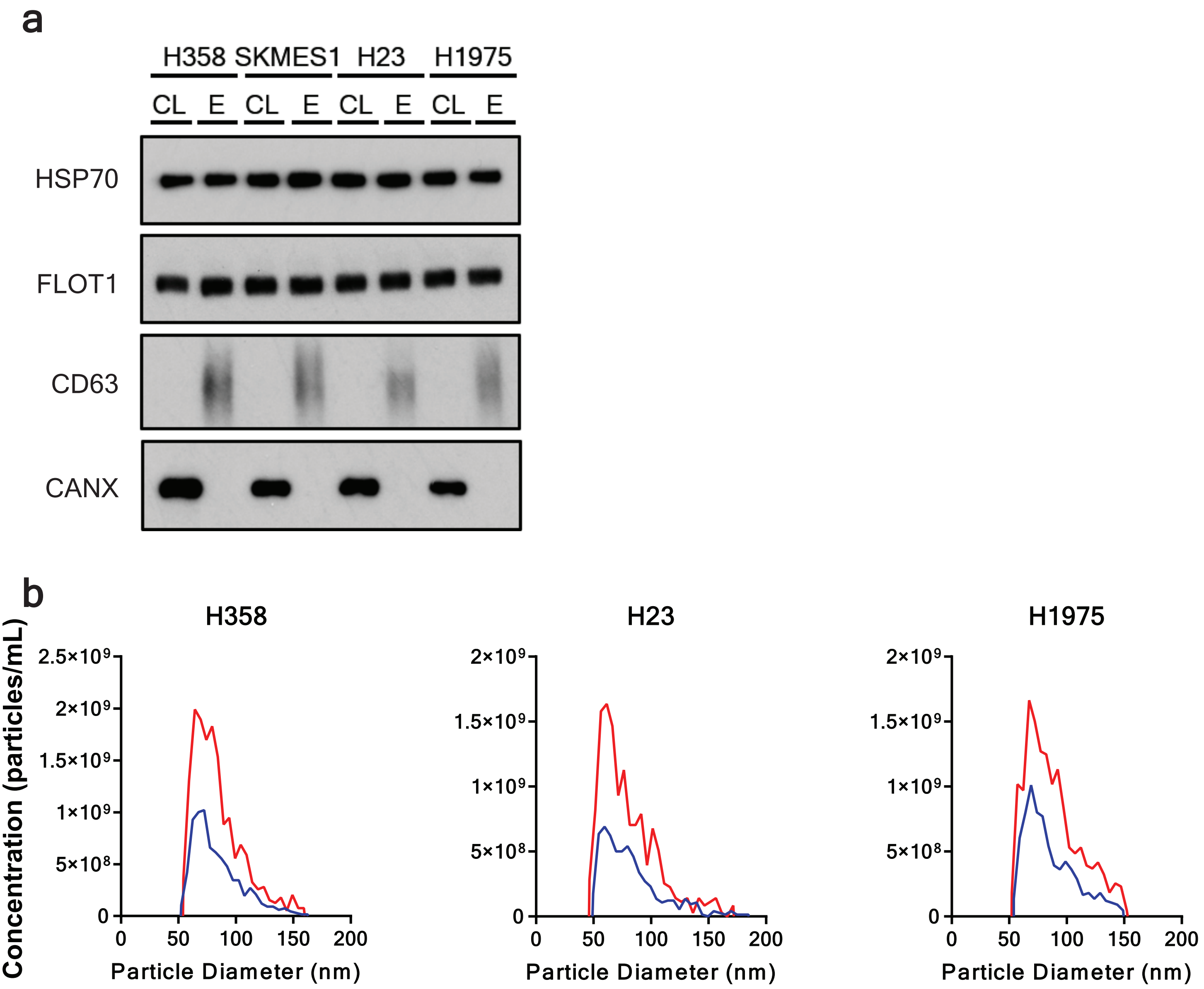

<u>Supplementary Figure 1:</u> Hypoxia increases sEV secretion from NSCLC cells. **a**, sEVs (E) isolated from NSCLC cell lines (CL) express canonical sEV markers HSP70, FLOT1, and CD63. The cell marker CANX is only found in cell lysates, not sEV lysates. **b**, Hypoxia increases sEV secretion from NSCLC cell lines.

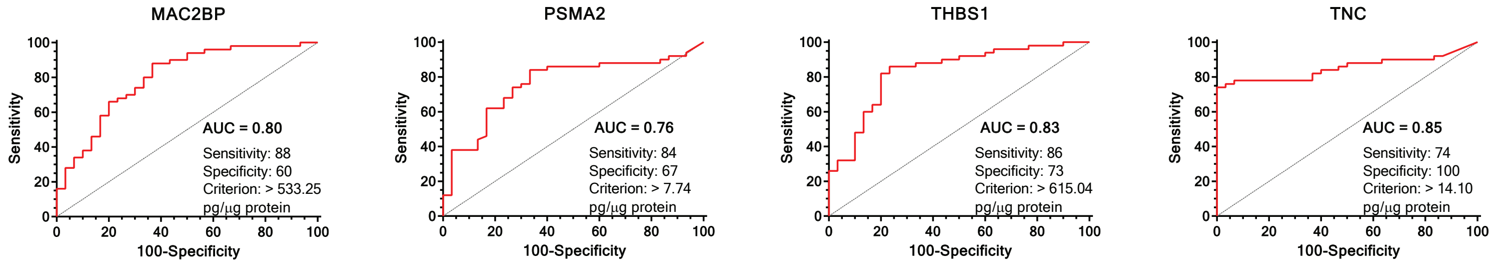

<u>Supplementary Figure 2:</u> Discovery cohort demonstrates sEV proteins are associated with disease progression in NSCLC patients. Individual ROC curves of each protein in the hypoxic sEV signature. Youden's index values are used to determine sensitivity and specificity.

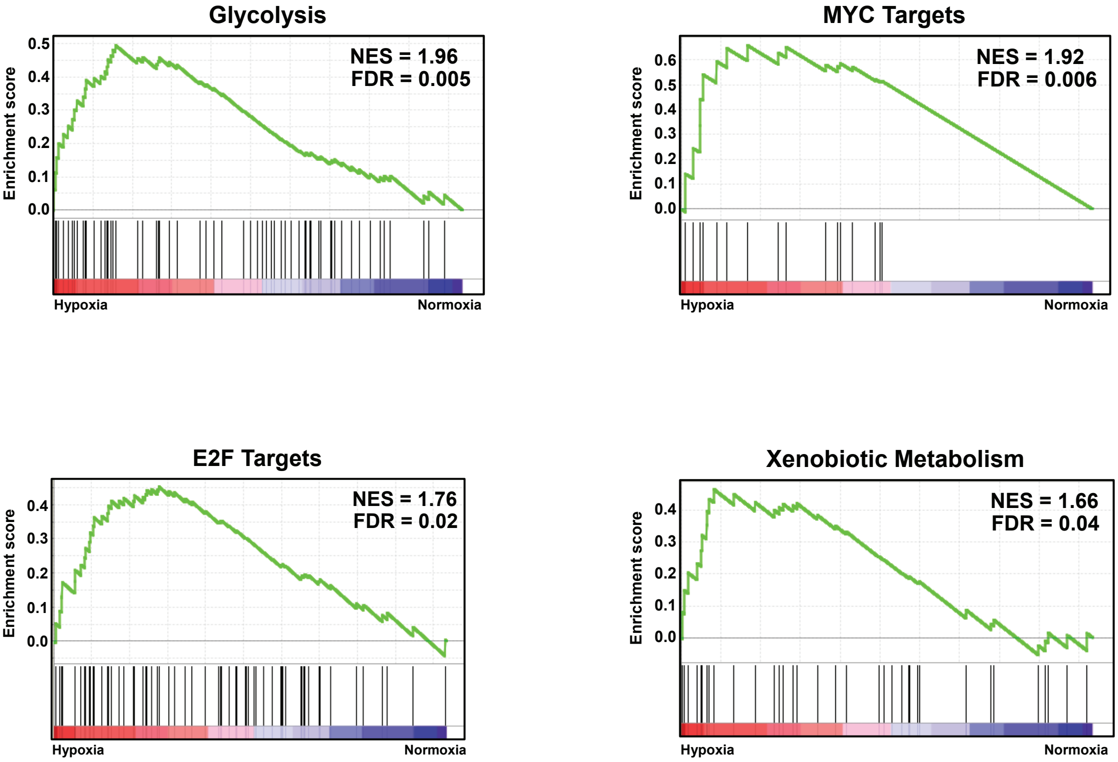

<u>Supplementary Figure 3:</u> Gene set enrichment analysis (GSEA) identified gene sets that were enriched in sEVs derived from hypoxic or normoxic SKMES1 cells. GSEA using the sEV protein expression dataset against hallmark gene sets reveals that hypoxic sEVs are enriched in proteins associated with glycolysis, MYC targets, E2F targets, and xenobiotic metabolism. NES - Normalised enrichment score.

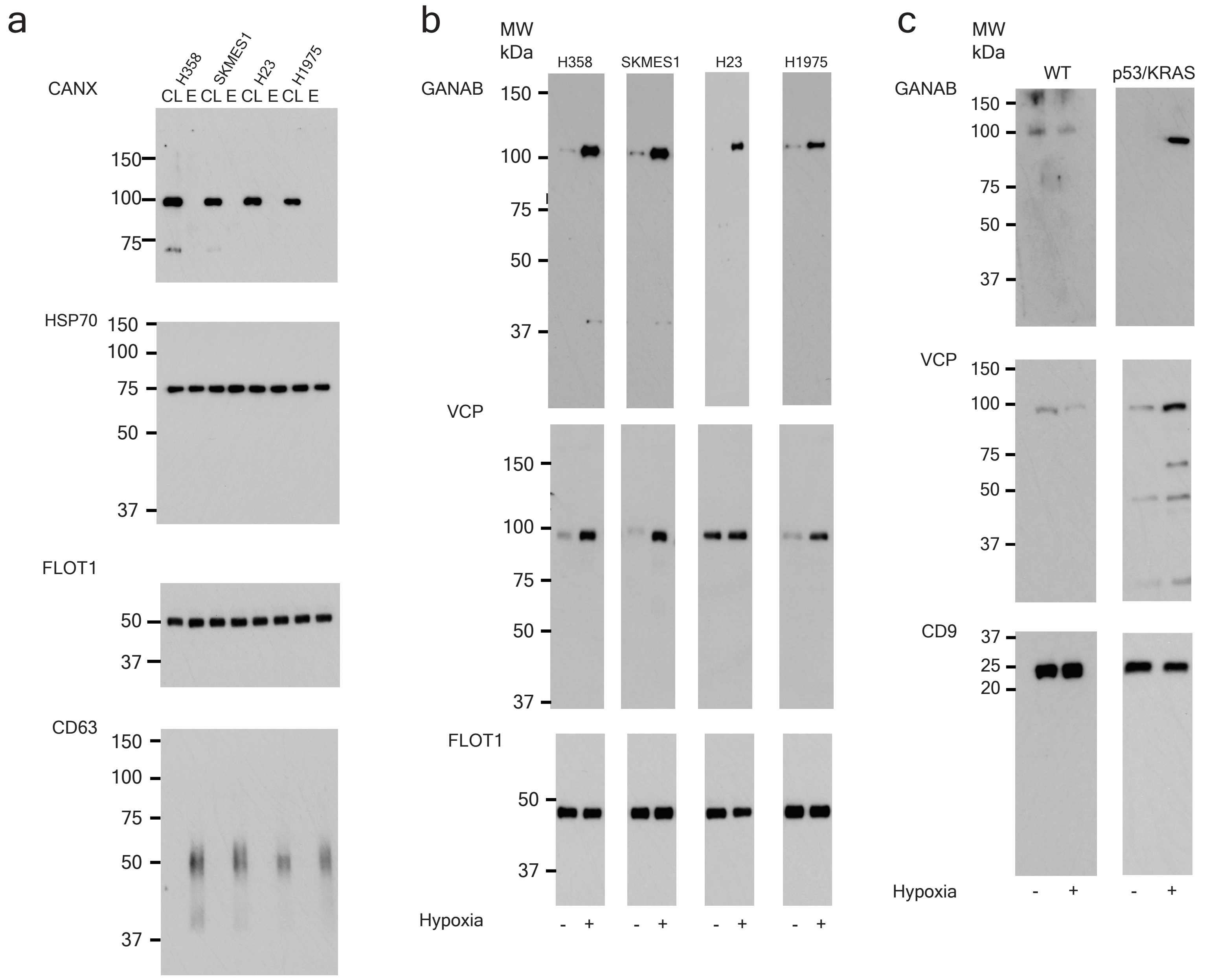

<u>Supplementary Figure 4:</u> Full length western blot images. **a**, western blot from Figure 1a & Supplementary Figure 1. **b**, western blot from Figure 1f. **c**, western blot from Figure 3c.

Characteristics	Slow Progression	Fast Progression
**Total samples**	30	50
**Age**		
Median	65.7	67.1
Range	50-83	49-90
**Gender**		
F	13 (43%)	9 (18%)
M	17 (57%)	41 (82%)

Supplementary Table 1:

Patient information of discovery cohort.

Characteristics	NSCLC Patients
**Total samples**	20
**Age**	
Median	68
Range	31-84
**Gender**	
F	4 (20%)
M	16 (80%)

Supplementary Table 2:

Patient information of confirmation cohort.
