# Supplementary material for: An epithelial-to-mesenchymal transition induced extracellular vesicle prognostic signature in non-small cell lung cancer
Source: Commun Biol. 2023 Jan 18;6:68. doi: 10.1038/s42003-022-04350-4 (PMC9849257; doi:10.1038/s42003-022-04350-4)
Supplement: Supplementary file 2 — Description of Additional Supplementary Files [file 42003_2022_4350_MOESM2_ESM.pdf]

Description of Additional Supplementary Files

File name: Supplementary Data 1

Description: Mass spectrometry data
