# Supplementary material for: An epithelial-to-mesenchymal transition induced extracellular vesicle prognostic signature in non-small cell lung cancer
Source: Commun Biol. 2023 Jan 18;6:68. doi: 10.1038/s42003-022-04350-4 (PMC9849257; doi:10.1038/s42003-022-04350-4)
Supplement: Supplementary file 4 — Reporting Summary [file 42003_2022_4350_MOESM4_ESM.pdf]

nature portfolio

Corresponding author(s): Andreas Möller
Last updated by author(s): Nov 3, 2022

Reporting Summary

Nature Portfolio wishes to improve the reproducibility of the work that we publish. This form provides structure for consistency and transparency in reporting. For further information on Nature Portfolio policies, see our Editorial Policies and the Editorial Policy Checklist.

Statistics

For all statistical analyses, confirm that the following items are present in the figure legend, table legend, main text, or Methods section.

n/a	Confirmed	
☐	☒	The exact sample size (*n*) for each experimental group/condition, given as a discrete number and unit of measurement
☐	☒	A statement on whether measurements were taken from distinct samples or whether the same sample was measured repeatedly
☐	☒	The statistical test(s) used AND whether they are one- or two-sided<br>*Only common tests should be described solely by name; describe more complex techniques in the Methods section.*
☐	☒	A description of all covariates tested
☐	☒	A description of any assumptions or corrections, such as tests of normality and adjustment for multiple comparisons
☐	☒	A full description of the statistical parameters including central tendency (e.g. means) or other basic estimates (e.g. regression coefficient) AND variation (e.g. standard deviation) or associated estimates of uncertainty (e.g. confidence intervals)
☐	☒	For null hypothesis testing, the test statistic (e.g. *F*, *t*, *r*) with confidence intervals, effect sizes, degrees of freedom and *P* value noted<br>*Give P values as exact values whenever suitable.*
☒	☐	For Bayesian analysis, information on the choice of priors and Markov chain Monte Carlo settings
☒	☐	For hierarchical and complex designs, identification of the appropriate level for tests and full reporting of outcomes
☒	☐	Estimates of effect sizes (e.g. Cohen's *d*, Pearson's *r*), indicating how they were calculated

Our web collection on statistics for biologists contains articles on many of the points above.

Software and code

Policy information about availability of computer code

Data collection	*Provide a description of all commercial, open source and custom code used to collect the data in this study, specifying the version used OR state that no software was used.*
Data analysis	*Provide a description of all commercial, open source and custom code used to analyse the data in this study, specifying the version used OR state that no software was used.*

For manuscripts utilizing custom algorithms or software that are central to the research but not yet described in published literature, software must be made available to editors and reviewers. We strongly encourage code deposition in a community repository (e.g. GitHub). See the Nature Portfolio guidelines for submitting code & software for further information.

Data

Policy information about availability of data

All manuscripts must include a data availability statement. This statement should provide the following information, where applicable:
- Accession codes, unique identifiers, or web links for publicly available datasets
- A description of any restrictions on data availability
- For clinical datasets or third party data, please ensure that the statement adheres to our policy

All data generated or analysed during this study are included in this published article (and its supplementary information files).

Human research participants

Policy information about studies involving human research participants and Sex and Gender in Research.

Reporting on sex and gender	We report on the sex of participants in the supplementary tables 2 and 3.
Population characteristics	Populations were selected as having NSCLC or not. Ages are described in supplementary tables 2 and 3.
Recruitment	Patients were recruited as part of a biobank through The Prince Charles Hospital (HREC/16/QPCH/281) or clinical trial ACTRN12611001283965 through the Peter MacCallum Cancer Centre.
Ethics oversight	This study was approved for the collection and use of all clinical samples by the human research ethics committees of The Prince Charles Hospital (HREC/16/QPCH/281), The University of Queensland (2017001730), the Peter MacCallum Cancer Centre (2008001483) and the QIMR Berghofer (P2180)

Note that full information on the approval of the study protocol must also be provided in the manuscript.

Field-specific reporting

Please select the one below that is the best fit for your research. If you are not sure, read the appropriate sections before making your selection.

☒ Life sciences ☐ Behavioural & social sciences ☐ Ecological, evolutionary & environmental sciences

For a reference copy of the document with all sections, see nature.com/documents/nr-reporting-summary-flat.pdf

Life sciences study design

All studies must disclose on these points even when the disclosure is negative.

Sample size	Sample size was chosen based on previous experience, in order to achieve a statistical power of approximately 80% (alpha=0.05).
Data exclusions	No data was excluded.
Replication	Experiments were repeated at least 3 times with distinct samples
Randomization	No randomization was performed.
Blinding	No blinding was performed.

Reporting for specific materials, systems and methods

We require information from authors about some types of materials, experimental systems and methods used in many studies. Here, indicate whether each material, system or method listed is relevant to your study. If you are not sure if a list item applies to your research, read the appropriate section before selecting a response.

Materials & experimental systems

n/a	Involved in the study	
☐	☒	Antibodies
☐	☒	Eukaryotic cell lines
☒	☐	Palaeontology and archaeology
☒	☐	Animals and other organisms
☐	☒	Clinical data
☐	☐	Dual use research of concern

Methods

n/a	Involved in the study	
☒	☐	ChIP-seq
☒	☐	Flow cytometry
☒	☐	MRI-based neuroimaging

Antibodies

Antibodies used	Calnexin (Cell Signalling Technology, 2679S), CD9 (Abcam, ab92726), CD63 (Abcam, ab8219), Flotillin-1 (BD Transduction Laboratories, 610821), HSP70 (Transduction Laboratories, 610608), TSG101 (Santa Cruz, sc-6037). Horseradish peroxidase (HRP)-conjugated secondary antibodies were purchased from Thermo Scientific.

Validation	The antibodies used for western blotting were validated either in Lobb et al. (DOI: 10.3402/jev.v4.27031) or Wen et al. (DOI: 10.1158/0008-5472.CAN-16-0868)

Eukaryotic cell lines

Policy information about cell lines and Sex and Gender in Research

Cell line source(s)	Human NSCLC cell lines H23, H358, H1975, and SKMES1; breast cancer MDA-MB-231; prostate cancer DU145, LNCaP, and PC-3; ovarian cancer CAOV3; pancreatic cancer MIA PaCa-2, PANC1; melanoma A375; and colorectal cancer HT29, SW620 were obtained from ATCC. Isogenic immortalized normal human bronchial epithelial cells (HBEC30KT) transformed with p53 knockdown (30KTp53), p53 knockdown and Kras v12 overexpression (30KTp53/KRAS) and p53 knockdown, Kras v12 overexpression, and LKB1 knockdown (30KTp53/KRAS/LKB1)14, H358-pMSCV (H358) and H358-pMSCV-ZEB1 (H358ZEB1), CALU1-pSRP (CALU1pSRP), and CALU1-pSRP-shZEB1 (CALU1shZEB1) were a gift from Dr. Jill Larsen
Authentication	Cell line authentication was carried out using short tandem repeat profiling
Mycoplasma contamination	All cell lines were repeatedly tested negative for mycoplasma in house at QIMR Berghofer
Commonly misidentified lines (See ICLAC register)	HT29 and PANC1 are used by us as surrogates for colorectal and pancreatic cancer, however, in combination with other cancer cells of the same cancer origin.

Clinical data

Policy information about clinical studies

All manuscripts should comply with the ICMJE guidelines for publication of clinical research and a completed CONSORT checklist must be included with all submissions.

Clinical trial registration	ACTRN12611001283965
Study protocol	Available online and through study manager Sarah Everitt<br>Radiation Therapy Peter MacCallum Cancer Centre Locked Bag 1, A'Beckett Street VIC 8006<br>61 3 96561111
Data collection	Key inclusion criteria Written informed consent provided.<br>Histologically or cytologically confirmed NSCLC.<br>Candidate for radical RT (60Gy) & concomitant chemotherapy (standard regimen determined by medical oncologist).<br>Stage I - III disease (TNM 7th edition).<br>ECOG performance status 0-1.<br>Key exclusion criteria<br><br>Received previous thoracic RT or a complete macroscopic excision of tumour.<br>Pregnancy (Women of child-bearing age to ensure adequate contraceptive measures).
Outcomes	not applicable as cancer stage at baseline and disease free survival were only used in this paper.

Dual use research of concern

Policy information about dual use research of concern

Hazards

Could the accidental, deliberate or reckless misuse of agents or technologies generated in the work, or the application of information presented in the manuscript, pose a threat to:

No	Yes	
☒	☐	Public health
☒	☐	National security
☒	☐	Crops and/or livestock
☒	☐	Ecosystems
☒	☐	Any other significant area

Experiments of concern

Does the work involve any of these experiments of concern:

No	Yes	
☒	☐	Demonstrate how to render a vaccine ineffective
☒	☐	Confer resistance to therapeutically useful antibiotics or antiviral agents
☒	☐	Enhance the virulence of a pathogen or render a nonpathogen virulent
☒	☐	Increase transmissibility of a pathogen
☒	☐	Alter the host range of a pathogen
☒	☐	Enable evasion of diagnostic/detection modalities
☒	☐	Enable the weaponization of a biological agent or toxin
☒	☐	Any other potentially harmful combination of experiments and agents
